# Predictors of flow state in performing musicians: an analysis with the logistic regression method

**DOI:** 10.3389/fpsyg.2023.1271829

**Published:** 2023-11-23

**Authors:** Laura Moral-Bofill, Andrés López de la Llave, Ma Carmen Pérez-Llantada

**Affiliations:** ^1^Escola Superior d’Estudis Musicals, ESEM, Taller de Músics, Barcelona, Spain; ^2^Department of Methodology of the Behavioral Sciences, Facultad de Psicología, Universidad Nacional de Educación a Distancia, Madrid, Spain

**Keywords:** challenges, flow state, feedback, skills, intrinsic motivation, performing musicians, goals, predictors

## Abstract

**Introduction:**

Flow state has been deemed a desirable state for performing musicians given its negative correlations with musical performance anxiety, its relationship to optimal performance, and its possible effect on creativity. In the field of music, there are a few studies that have assessed intervention programmes to promote flow state in performing musicians with varying results in terms of their success. The flow condition-experience model proposes three components that would be the conditions for flow state to occur and six components that describe the experience of being in a flow state. In addition, within the vast academic literature on this experience, other factors that could influence its occurrence have been proposed. The main objective of this research was to detect which are the most suitable predictors from a set of independent variables collected to distinguish performing musicians with a high flow level.

**Methods:**

A binary logistic regression analysis was carried out with data from 163 musicians aged between 18 and 65. Independent variables were introduced in the analysis: skill-challenge balance, clear goals and clear feedback (condition-experience model); and also, gender, age, dedication, (musical) style, musical instrument and (performing) situation.

**Results:**

The results showed that the three conditions of the condition-experience model and the situation variable had positive associations with flow state. The model explained 78% of the variance of the dependent variable and obtained a 90.8% correct classification rate.

**Discussion:**

These variables seem to contribute most to a high flow level, and the importance of keeping in mind the intrinsic reasons why performers dedicate themselves to music is emphasised. The results and their implications for the training of performing musicians are discussed. Future lines of research are proposed, as well as collecting data on personality-related variables to introduce them into the regression model.

## 1 Introduction

The flow experience was described by Csikszentmihalyi (1975) to explain why people engage in activities for no other reason than the activity itself, without extrinsic rewards, and persist in those activities. In the field of Sport Psychology it is a widely researched construct that has contributed to developing athletes’ psychological skills to optimise their enjoyment and performance (Jackson and Csikszentmihalyi, 1999; Norsworthy et al., 2017; Jackman et al., 2019). But there has also been research in other fields, such as work (Csikszentmihalyi and Lefevre, 1989; Bryce and Haworth, 2002; Eisenberger et al., 2005; Peifer and Wolters, 2021), education (Carli et al., 1988; Bakker, 2005; Rathunde and Csikszentmihalyi, 2005), creativity (Csikszentmihalyi, 1996; Csikszentmihalyi and Rich, 1998), leisure (Lefevre, 1988; Schallberger and Pfister, 2001), the arts (*cf.* Harmat et al., 2021), human-computer interaction (Triberti et al., 2021), high abilities (Moral-Bofill et al., 2020a), or physical/cognitive rehabilitation in patients with neurological diseases (*cf.* Ottiger et al., 2021).

In the field of music, there are increasingly more studies around musical education and performance (Custodero, 2002, 2005; Fritz and Avsec, 2007; Sinnamon et al., 2012; Fullagar et al., 2013; Marin and Bhattacharya, 2013; Wrigley and Emmerson, 2013; Iusca, 2015; Cohen and Bodner, 2019a,b, 2021; Moral-Bofill et al., 2020b, 2022a,b; Moral-Bofill, 2021, 2022) (see also Chirico et al., 2015; Tan and Sin, 2021). For example, flow has been linked to creativity, improving creative composition activities (Byrne et al., 2003; MacDonald et al., 2006). For performing musicians, studies show that experiencing flow can contribute to a greater enjoyment of the performance activity and a reduction in music performance anxiety (MPA) (Kirchner et al., 2008; Sinnamon et al., 2012; Fullagar et al., 2013; Wrigley and Emmerson, 2013; Cohen and Bodner, 2019a,b, 2021; Spahn et al., 2021; Moral-Bofill et al., 2022a,b). It is also an important source of motivation for musicians to remain involved in music (Woody and McPherson, 2010).

As with sport, the need for musicians to develop psychological self-regulation skills, and for training to focus not only on purely technical-performance skills, has been considered (Brodsky, 1996; Williamon, 2004; Clark and Williamon, 2011; Wrigley and Emmerson, 2013; Cohen and Bodner, 2019a; Spahn et al., 2021; Moral-Bofill et al., 2022a,b). Since performing musicians are exposed to physical and psychological stress that can lead to disorders and health problems, it would be important to implement appropriate interventions to help make their musical career rewarding and sustained over time (Kenny and Ackermann, 2016). It has been suggested that interventions should target skills training related to flow factors and the personality characteristics of the individual (such as good preparation, task concentration, coping strategies, goal setting, motivational exercises, confidence building and activation management) (Swann, 2016). Recent research that assessed an intervention programme applied to performing musicians showed that the programme had a positive and statistically significant influence on both the musicians’ overall flow level and on two of the components of flow: sense of control and loss of self-consciousness. The conclusion was that programmes whose designs include a combination of all the techniques and methods that were used in the programme and that come from psychology could be useful to treat or prevent the problem of MPA, and could also facilitate flow state, greater enjoyment while performing and potentially better performance quality (Moral-Bofill et al., 2022b). Flow theory contributes to the understanding of participation in activities such as music, but also to finding strategies for musical educational practise and for the improvement of skills (Fullagar et al., 2013), including technical and expressive training (Custodero, 2002). As an unequivocally positive performance-related experience, it could be key to encouraging educators to promote it in students (Sinnamon et al., 2012). It is related to the musical performance of classical music students (Clark et al., 2014) and university students during exams (Iusca, 2015). It is also related to the self-perceived musical quality after a performance, which could increase self-confidence for future performances and improve the musician’s ability to cope with MPA (Spahn et al., 2021).

### 1.1 Flow differences between performing musicians

There were mixed results when regarding possible differences in the flow experience between performing musicians.

Some studies have found no differences regarding sex (or gender) (Marin and Bhattacharya, 2013; Wrigley and Emmerson, 2013; Cohen and Bodner, 2019b, 2021; Spahn et al., 2021). However, others have found that flow tendency is higher in male than in female music students (Habe et al., 2019, 2021), or that males obtain higher flow state scores than females (Moral-Bofill et al., 2019).

In terms of age, there is no evidence of an association with flow experience in different areas (Delle Fave et al., 2011), however, it has been shown to be a statistically significant predictor of flow experience in musicians (Spahn et al., 2021). It has also been positively associated with flow disposition in professional orchestral musicians (Cohen and Bodner, 2021). However, this could be a spurious effect and the result of greater musical experience. In fact, in the latest study, a high correlation was found between age and the years musicians had been performing.

In terms of the relationship musicians have with musical activity (e.g., students, professionals or amateurs), lower flow disposition scores have been found in students compared to professionals (Cohen and Bodner, 2019a). It was also found that students had a lower flow state compared to professional and amateur musicians (Moral-Bofill et al., 2019). In contrast, flow disposition was not found to be higher in elite musicians compared to amateur musicians (Sinnamon et al., 2012), nor with flow experience measures (Kutepova-Bredun, 2018; Spahn et al., 2021).

According to the style of music, it was found that jazz/modern and traditional musicians showed higher flow state scores than classical tradition musicians. It was suggested that classical musicians might be affected by the more academic and structured environment of their studies in order to experience a higher flow state (Moral-Bofill et al., 2019). Furthermore, research results suggest that tests do not promote or reinforce effective levels of self-efficacy and confidence, and in most students they may inhibit the true level of learning achieved (Wrigley and Emmerson, 2013).

There are also findings that showed that flow disposition was higher in musicians who performed in a group compared to those who performed individually (Habe et al., 2019, 2021), and it was also higher in the orchestra’s main instrumentalists compared to the other musicians in the orchestra (Cohen and Bodner, 2021), and it was also shown that adolescent contestants performing popular music had higher flow scores than contestants performing classical music (Bullerjahn et al., 2020).

Finally, there are no links between instrument type and flow state in music students during their participation in examinations (Wrigley and Emmerson, 2013), nor in the flow experience among different categories of classical music instrumentalists (Spahn et al., 2021). However, percussionists have been found to experience a higher flow disposition compared to string section players (Cohen and Bodner, 2021); and also a higher flow state compared to violinists, wind players and pianists (Moral-Bofill et al., 2019).

### 1.2 Flow triggers

On the other hand, accumulated research on flow shows that the most universal precondition for triggering the flow response is the balance between the perceived challenges in a situation and the individual’s skills to engage. However, the relationships between these variables may be moderated by situational and personal factors (*cf.* Barthelmäs and Keller, 2021). For example, in terms of situational factors, it has been shown that the relationship between optimal challenge (skill-challenge balance) and enjoyment was greater in activities that were intrinsically motivated and in the context of goal-directed activities (Abuhamdeh and Csikszentmihalyi, 2012). For this reason, it has been suggested that the optimal challenge concept would predict enjoyment in the context of goal-directed leisure activities, such as sports and games, rather than in contexts where performance outcomes could have greater consequences (Abuhamdeh, 2021). In line with this argument, research with students on performance in different types of tasks showed that when skills and demands were in balance relatively high flow scores were obtained if the activity’s importance was assessed as relatively low. However, if the activity’s perceived importance was relatively high, flow scores were higher when skills exceeded demands (Engeser and Rheinberg, 2008). One might think, for example, of examinations or evaluative or public exhibition situations. Another study also suggests that the activity’s importance may partially explain differences in the flow experience between work and leisure contexts (Engeser and Baumann, 2016). In addition, it has also been considered that the loss of self-consciousness (as part of the flow experience) may be hindered in situations that increase people’s self-consciousness (Barthelmäs and Keller, 2021).

In terms of personal factors, different studies have linked the balance between skills and demands to different personality characteristics. For example, it was linked to higher flow scores in people characterised by a low level of fear of failure. While people who reported a high level of fear of failure experienced more flow when their skills exceeded the demands (Engeser and Rheinberg, 2008). Furthermore, flow has also been shown to be negatively linked to neuroticism and positively linked to responsibility, but not to intelligence (Ullén et al., 2012). Specifically in musicians, flow experience correlated positively with extraversion and negatively with neuroticism scores (Heller et al., 2015). It has also been suggested that people with high internal locus of control scores may enjoy the activity more when faced with challenges and reach flow states more easily (Keller and Blomann, 2008; Mosing et al., 2012). But also, the need for achievement (Eisenberger et al., 2005), mental tenacity (Crust and Swann, 2013), self-control (Kuhnle et al., 2012), the quest for novelty and persistence (Teng, 2011), or people with a high level of action orientation (Keller and Bless, 2008; Baumann et al., 2016) have shown positive relationships with flow.

In addition to the balance between challenge and skill, the theory distinguishes two further conditions that would bring about flow state. On the one hand, having clear goals and, on the other, clear feedback. Three conditions are necessary for a person to be able to assess the level of challenges they face and the necessary skills they need to engage (Nakamura et al., 2019). While flow state, i.e., the subjective experience of flow would be characterised by (a) Concentration on the task, (b) Merging of action and awareness, (c) Loss of self-consciousness, (d) Sense of control, (e) Transformation of time; and (f) Autotelic experience (Nakamura et al., 2019).

The relationships between the conditions and the components of the flow experience are represented in the condition-experience model. Together, the three conditions should lead to a flow experience characterised by the six components of flow state. The model shows how the flow experience conditions are interconnected and, also, that it would be difficult to be in a flow state if any of these conditions were absent (see Nakamura et al., 2019).

It has been suggested that it is important to continue studying the factors that precede the flow experience. Apart from the balance between challenges and skills, the rest of the proposed factors have partial or only theoretical empirical support (Peifer and Engeser, 2021).

### 1.3 Flow operationalization

Another relevant aspect within the theory is the need to operationalise flow state as an optimal state. As an optimal state, it would also be expected to lead to exceptional performance, as an intrinsically rewarding state it would lead to greater commitment to the activity over time; and that means a greater possibility to do things, to act and to be creative (Csikszentmihalyi, 1988). In other words, flow is a source of intrinsic motivation, which any skill requiring complex behaviour and high concentration depends on. This means that cognitive skills alone do not guarantee successful development unless a person enjoys or likes what they are doing (Csikszentmihalyi, 1988).

Furthermore, according to Csikszentmihalyi (1988), the flow experience and its motivating force is reduced when all the components of the positive flow state experience do not occur together. This consideration suggests that flow state can be operationalised as a discrete construct. It has been noted that flow state should be properly operationalised as a relatively rare optimal state of consciousness in everyday life, which is intrinsically rewarding and differentiated from the conditions that trigger it. As an optimal state of consciousness, it would be understood as a discrete construct, i.e., one is or is not in a flow state (even if measured by instruments with ordinal-type scales) (Abuhamdeh, 2020). Studies that have conducted analyses using non-linear regression techniques have shown that there are drastic and discontinuous changes in the flow experience (Ceja and Navarro, 2012), and that the fit indices of the non-linear model are better than the indices of the linear model for all participants (Bricteux et al., 2017). These results suggest that the appearance and disappearance of flow state happens suddenly and should be deemed a presence-absence phenomenon rather than a matter of degree. This would imply the use of other types of scales to measure it more accurately (Bricteux et al., 2017).

Indeed, it has been considered that questionnaires measuring flow based on multidimensional models need to address some limitations (Moneta, 2021). One of the most widely used scales consistent with the nine-component flow model is the FSS-2 (Jackson and Csikszentmihalyi, 1999; Jackson and Eklund, 2002, 2004). This scale is widely used in the sport context, and has also been adapted to the field of musical performance (Sinnamon et al., 2012; Wrigley and Emmerson, 2013). However, these scales do not consider the condition-experience model (see Nakamura et al., 2019), and contradict the distinction between the antecedent of flow and the flow state experienced. This is a relevant consideration, since the balance between challenge and skill has consistently been shown to be an antecedent of flow in regression studies (Moneta, 2021). In addition, both experimental and correlational studies have shown the importance of the perceived fit between the skills and challenges of the task to experience flow (see Barthelmäs and Keller, 2021). However, a recent adaptation of the FSS-2 to Spanish in a population of musicians has taken into account the distinction between conditions and experience (Moral-Bofill et al., 2020b). In addition, there is also previous work with musicians that adapted the FSS-2 using the item with the highest factor loadings from each of the six scales measuring flow state (Fullagar et al., 2013). However, although the Moral-Bofill et al. (2020b) scale assumes the condition-experience model, it is not exempt from the limitation that an 11-point scale is used, which makes it difficult to detect the cut-off point at which flow would be reached. If the scores on the six experience components are not homogeneously high, it is difficult to conclude that flow state has been fully achieved.

### 1.4 Aims and research questions

In light of the above considerations, there may be a way to address such a limitation. If a scale that measures flow state without including the three “condition” components (for example, see Moral-Bofill et al., 2020b) is available, the variable flow state can be categorised into a dichotomous variable by establishing a cut-off point from which scores are considered to be high. Although it would not be a guarantee of the presence of flow state, it is likely to be closer to that state.

The main objective of this research was to try to detect which predictors can be considered the most suitable for identifying performing musicians with high flow levels during musical performance by applying the logistic regression (LR) method. Flow theory establishes antecedent variables with more or less empirical support (Peifer and Engeser, 2021). The variable with the greatest empirical support is the skill-challenge balance, which was introduced into the model, but the other two conditions were also explicitly taken into account (clear goals and clear feedback). On the other hand, other variables studied in the field of performing musicians, which do not always present homogeneous results, were taken into account. On the one hand, variables that could be considered situational or related to specific situations (such as the context in which it is played, the style of music, the relationship with the musical activity, or the musical instrument), on the other hand, sociodemographic variables such as age and gender.

## 2 Method

### 2.1 Participants

The study began with 323 musicians from Spain who responded to the form that collected data on the variables covered by the research. Of these 323 cases, 164 musicians were selected on the basis of their score in the flow state variable (high and low), as detailed in the data analysis section. Subsequently, during the inspection of this new database, one case was eliminated because it was the only representative of the “I prefer not to answer” category of the gender variable; thus, the total number of study participants was *N* = 163.

The musicians were performers with a minimum of 2 years’ experience playing their main instrument. The mean age was 36.33 years (SD = 12.32), with a range of 47 between 18 and 65 years. 41.7% were men (mean age = 37.03; SD = 12.02) and 58.3% were women (mean age = 35.82; SD = 12.58). Regarding dedication, 16.6% were music students, 25.8% amateurs and 57.7% professionals. As for the style of music, 65% were musicians with a classical-contemporary style profile (including those specialising in early music) and 35% were traditional and modern style (including flamenco and jazz). The type of situation in which the musicians had performed was also collected. 76.7% of the participants had responded to the form after performing a concert (concert situation) and 23.3% after playing in an individual or informal situation (which we labelled as free situation). The time between the performance situation and the completion of the form was less than 1 week. The main musical instrument was also collected and categorised by instrument families. Descriptive information was collected on the type of centre where they studied or were still studying if they were students (study centre). To see the characteristics of the 163 participants in terms of the categorical variables covered, see Table 1.

**Table 1:** Characteristics of the participants in terms of the categorical variables considered.

Variable	Categories	Percentages %
Gender	Men	41.7
	Women	58.3
Style	Classic and contemporary	65
	Traditional and modern	35
Musical instrument	Wind	27.6
	Piano	17.8
	Percussion	7.4
	Singer	14.7
	Plucked strings	7.4
	Bowed strings	16.6
	Other	8.6
Situation	Concert situation	76.7
	Free situation	23.3
Dedication	Student	16.6
	Professional	57.7
	Amateur	25.8
Study centre	Professional conservatoire	22.1
	Superior conservatoire	40.5
	Music school	16.6
	Other	20.9

### 2.2 Measuring instruments

A form was created using the Google Forms tool. It was designed with: (a) a section for sociodemographic data collection and some relevant questions about the musical activity of the participating musicians, (b) a section for the validated scale “Flow State for Musical Performers,” and (c) three *ad hoc* statements to assess each of the three antecedent variables or conditions of flow state.

In the first section, data was collected on age, gender, dedication, musical style, musical instrument, performance situation and study centre (see Table 1).

The second section included the “Flow State for Musical Performers” scale (Moral-Bofill et al., 2020b). This scale consists of 24 items measuring flow state. It consists of six subscales, each with four conceptually different items: action-awareness merging; concentration on the task; sense of control; loss of self-consciousness; transformation of time; and autotelic experience. To assess the degree of agreement with the formulation of each item, a Likert scale from 0 to 10 points is used, where 0 is strongly disagree and 10 is strongly agree. The scores for each of the 6 subscales can be obtained separately, as well as the overall flow state scale, which can adopt values between 0 and 240. Reliability indices with Cronbach’s Alpha are above 0.80 for all subscales and 0.92 for the overall flow state scale (Moral-Bofill et al., 2020b). To respond to the scale, the musician has to choose which performance situation they are taking as a reference to answer. As the authors of this scale indicate, the ideal way to answer the scale is at the end of the chosen situation or shortly after (Moral-Bofill et al., 2020b).

The third section included the three variables that would be the antecedents of flow state. Three scales from 0 to 10 were created to measure the degree of agreement with three statements corresponding to the three conditions for flow state to occur (condition-experience model, Nakamura et al., 2019). That is, skill-challenge balance (My skills were at the level of the challenge I was facing), clear goals (My objectives were clearly defined), and clear feedback (I was aware of how I was doing). In the case of the statement corresponding to the skill-challenge balance variable, it was taken into account that the perceived fit between the challenges would be measured (Barthelmäs and Keller, 2021).

### 2.3 Procedure

The research was endorsed by the Department of Behavioural Sciences Methodology of the Faculty of Psychology at the National University of Distance Education (UNED, in its Spanish acronym). Furthermore, the study was conducted in accordance with the latest Helsinki Declaration [World Medical Association (WMA), 2022]. An online form was shared with musicians from different music schools in Spain. Participation consisted of completing the form that was programmed using the Google Forms tool (see Instruments section) and recipients were informed that participation was anonymous and voluntary.

### 2.4 Data analysis

First, the database was examined for the possible presence of missing and atypical data. No missing data was identified as the form was adjusted so that no item was left unanswered. Atypical cases were kept within the analysis because the values were within the possible range that the variables can assume.

In order to choose the predictor variables that could be the most appropriate for identifying performing musicians with high flow levels, a Logistic Regression (LR) analysis was carried out. As a preliminary step for carrying out the LR analysis, the continuous quantitative variable flow state was transformed into a dichotomous categorical variable (low flow/high flow). To do so, musicians with scores below the first quartile (Q1) and above the third quartile (Q3) were selected. Specifically, a low flow musician was defined as a musician with flow state scores equal to or below 149 (Q1) and a high flow musician was defined as a musician with flow state scores equal to or above 197 (Q3). The mean score of Q1 was 127.75 (SD = 21.57) and the mean score above Q3 was 212.75 (SD = 11.36). Thus, the new dichotomized categorical variable cat_flow was made up of the low flow category (coded as 0) with 80 participants and by the high flow category (coded as 1) with 83 participants; the rest of the cases that obtained scores between the two quartiles Q1 and Q3 were not taken into account in the analyses.

In order to assess the suitability of applying logistic regression (LR), the assumptions of: (a) linearity between the quantitative independent variables and the logit of the dependent variable and (b) absence of multicollinearity and uniqueness among the independent variables were verified. In order to verify that the relationship between the logit of the response variable and each predictor variable or continuous independent variable was linear, the Box-Tidwell test was carried out. As a first step we included the interactions between the continuous predictors and their logs in the model. The interaction terms were not statistically significant in any case, so each of the original continuous independent variables were linearly related to the logit of the dependent variable. The absence of multicollinearity and uniqueness was verified by calculating bivariate correlations and the tolerance indices and variance inflation factor (VIF) of the variables involved in the regression analysis. The correlation matrix did not show values greater than 0.80. It is considered that a correlation coefficient greater than 0.80 can cause collinearity problems. The tolerance indices were greater than 0.10 (between 0.37 and 0.95) and those for VIF were less than 10 (between 1.04 and 2.70). Therefore, according to these two indices, no multicollinearity problems were observed. The verification of the assumptions indicated the suitability of applying the LR except for the assumption of independence between observations. This assumption is related to the data collection process, specifically, when a simple random sample is taken.

In order to describe the characteristics of the participating musicians, descriptive statistics were obtained from the different categories of the qualitative variables collected (see Table 1), as well as from the three quantitative independent variables skill-challenge balance (balance), clear goals (goals), and clear feedback (feedback) (see Tables 2, 3).

**Table 2:** Descriptive statistics of the independent variables *balance*, *goals*, and *feedback*, *N* = 163.

	Minimum	Maximum	Mean	SD
Balance	1	10	7.90	2.106
Goals	2	10	8.04	1.946
Feedback	1	10	7.88	2.099

**Table 3:** Descriptive statistics of the independent variables *balance*, *goals*, and *feedback*, depending on the group (*low flow* [0], *n* = 80; *high flow* [1], *n* = 83) of the *cat_flow* variable.

	Cat_flow	Mean	SD
Balance	0	6.40	1.972
	1	9.35	0.833
Goals	0	6.74	1.805
	1	9.29	1.054
Feedback	0	6.60	1.907
	1	9.11	1.440

As a first step of the logistic regression analysis, age, gender, dedication, musical instrument, style, situation and the three variables, which are considered the necessary conditions for flow state to occur: balance, goals and feedback, were introduced as predictor variables. The type of independent variables that can be introduced into the LR analysis can be quantitative or qualitative variables, but since LR proceeds quantitatively, qualitative or nominal variables were previously coded. For the dichotomous variables gender, style and situation, they were coded as male (0) female (1); classic-contemporary (0) traditional-modern (1); and concert situation (0) free situation (1) respectively. In the case of the dedication and musical instrument variables, a special treatment was provided by creating dummy variables; 3–1 and 7–1 dichotomous variables, respectively (Silva and Barroso, 2004). Based on the results obtained in the first step, a second analysis was carried out by introducing the variables with predictive ability: situation, balance, goals and feedback.

To check the model fit, the Nagelkerke R2 coefficient was calculated, which determines the degree of association between the variables involved in the model. In addition, in order to have a measure of its predictive ability, a classification table was prepared to obtain sensitivity and specificity. The table is double entry and crosses the variable’s observed values with those predicted by the model considered for each of the cases. A model with good predictive ability should have high values for both sensitivity and specificity. Sensitivity refers to its ability to detect as positive the cases that possess the characteristic (high flow), while specificity refers to the ability to correctly discriminate cases that do not possess the characteristic (Silva and Barroso, 2004). All analyses were carried out with SPSS 24.0.0. and RStudio 2023.06.1.

## 3 Results

Table 2 shows the descriptive statistics for the three independent variables that are considered as conditions for flow state to occur: balance, goals and feedback. Table 3 shows the descriptive statistics depending on the group (low flow = 0; high flow = 1) for the categorised flow variable (cat_flow).

The results of the LR are presented below. First, the nine variables were entered into the analysis with the Enter method. This model correctly classified 91.4% of the musicians into their corresponding group (high flow or low flow). Table 4 shows the coefficients of the variables in the equation of this model. However, the Wald test showed that only four of the nine variables, balance, goals, feedback, and situation, were statistically significant predictors of group membership. Gender, age, dedication, musical instrument and style of music were not statistically significant predictors.

**Table 4:** Coefficients of the nine variables in the equation.

		*B*	SD	Wald	df	*p*	Exp(B)	95% C.I. for EXP(B)	
								Lower	Upper
Balance	2.026	0.491	17.047	1	0.000	7.582	2.898	19.835	Goals	0.698	0.311	5.051	1	0.025	2.009	1.093	3.693	Feedback	0.779	0.312	6.224	1	0.013	2.178	1.182	4.015	Gender	1.242	0.889	1.951	1	0.163	3.463	0.606	19.787	Age	0.024	0.036	0.449	1	0.503	1.025	0.954	1.100	Dedication			3.572	2	0.168				Dedication (1)	0.160	1.177	0.019	1	0.892	1.174	0.117	11.788	Dedication (2)	−1.606	0.989	2.640	1	0.104	0.201	0.029	1.393	Instrument			6.563	6	0.363				Instrument (1)	0.240	1.620	0.022	1	0.882	1.272	0.053	30.424	Instrument (2)	3.074	2.346	1.717	1	0.190	21.637	0.218	2149.521	Instrument (3)	1.090	1.410	0.597	1	0.440	2.973	0.187	47.186	Instrument (4)	−1.328	1.850	0.515	1	0.473	0.265	0.007	9.955	Instrument (5)	0.698	1.294	0.291	1	0.590	2.010	0.159	25.415	Instrument (6)	−1.040	1.327	0.614	1	0.433	0.354	0.026	4.760	Style	0.516	0.887	0.338	1	0.561	1.675	0.294	9.534	Situation	2.618	1.160	5.094	1	0.024	13.706	1.411	133.088	Constant	−30.414	6.723	20.467	1	0.000	0.000		

Therefore, the model was re-estimated with the four statistically significant predictor variables: balance, goals, feedback and situation.

The omnibus test with the chi-square statistic showed a statistically significant model (*χ*2 = 144.67; 4 df; *p* < 0.001). The statistical significance of the Beta (B) coefficients of the four variables indicated that they were significantly different from zero, and, therefore, that they contributed significantly to predicting the outcome (see Table 5).

**Table 5:** Coefficients of the four variables in the equation.

										95% C.I. for Exp(B)
		B	SD	Wald	df	*p*	Exp(B)	Lower	Upper
Situation	2.380	0.831	8.201	1	0.004	10.806	2.120	55.097	Balance	1.480	0.343	18.636	1	0.000	4.394	2.244	8.605	Goals	0.554	0.238	5.247	1	0.022	1.723	1.082	2.745	Feedback	0.500	0.171	8.511	1	0.004	1.648	1.178	2.306	Constant	−21.259	3.694	33.112	1	0.000	0.000		

**Figure 1:**
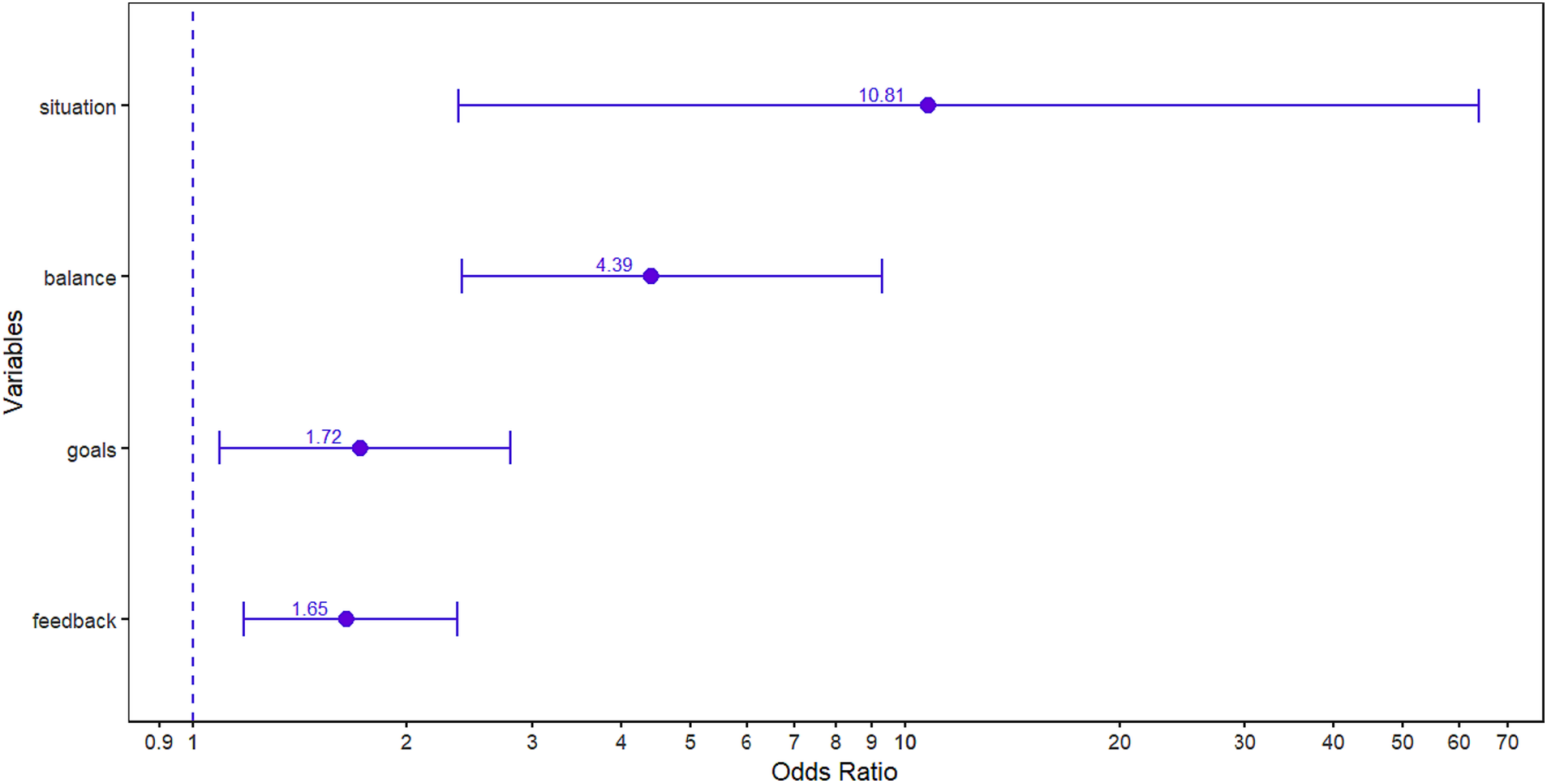
Odds Ratio of the four predictor variables of the regression model. It is necessary to consider that *B* = 0 is equivalent to OR = 1; therefore, OR = 1 means that the independent variable in question would not be associated with the probability of *high flow*.

On the one hand, the coefficient *B* = 2.380 of the situation variable showed a positive association with cat_flow. This association indicates a direct relationship between free situation (coded 1) and high flow (coded 1). The value of Exp(B) = 10.806 would indicate that the odds of obtaining high flow increase when performing in a free situation with respect to the odds of obtaining high flow when performing in a concert situation, once the possible influence of the rest of the independent variables on the odds of experiencing high flow has been controlled. Specifically, it would indicate that the odds of high flow with a free performance situation are 10.806 times the odds of high flow with a concert performance situation. On the other hand, the balance variable showed a positive association with cat_flow. Specifically, it indicated that the higher the balance score, the higher the odds of obtaining a high flow score. The coefficient B expresses the change in Exp(B) when the variable increases by one unit and the rest of the variables remain constant. Therefore, the value of *B* = 1.480 indicated that each additional balance score produced an increase in the odds ratio (OR) of obtaining high flow that amounted to Exp(B) = 4.394 (for example, a musician with a balance score of 2 would have an OR of obtaining high flow of 4.394 times greater than a musician who only scores 1 – it should be noted that a characteristic of the exponential function is that the function is increasing and increases very rapidly as the independent variable score increases). Similarly, the goals and feedback variables showed a positive association with cat_flow. Specifically, they indicated that the clearer the goals and feedback, the higher the odds of obtaining a high flow score. The value of *B* = 0.544 indicated that each higher goals score produced an increase in the OR of obtaining high flow amounting to Exp(B) = 1.723. In the same way, the value of *B* = 0.500 indicated that each additional feedback score produced an increase in the OR of obtaining high flow amounting to Exp(B) = 1.648 (see Table 5; Figure 1).

As for the goodness-of-fit of the model, the Nagelkerke’s R2 value was 0.78. Thus, the independent variables entered into the regression model explained 78% of the variance of the dependent variable cat_flow.

**Figure 2:**
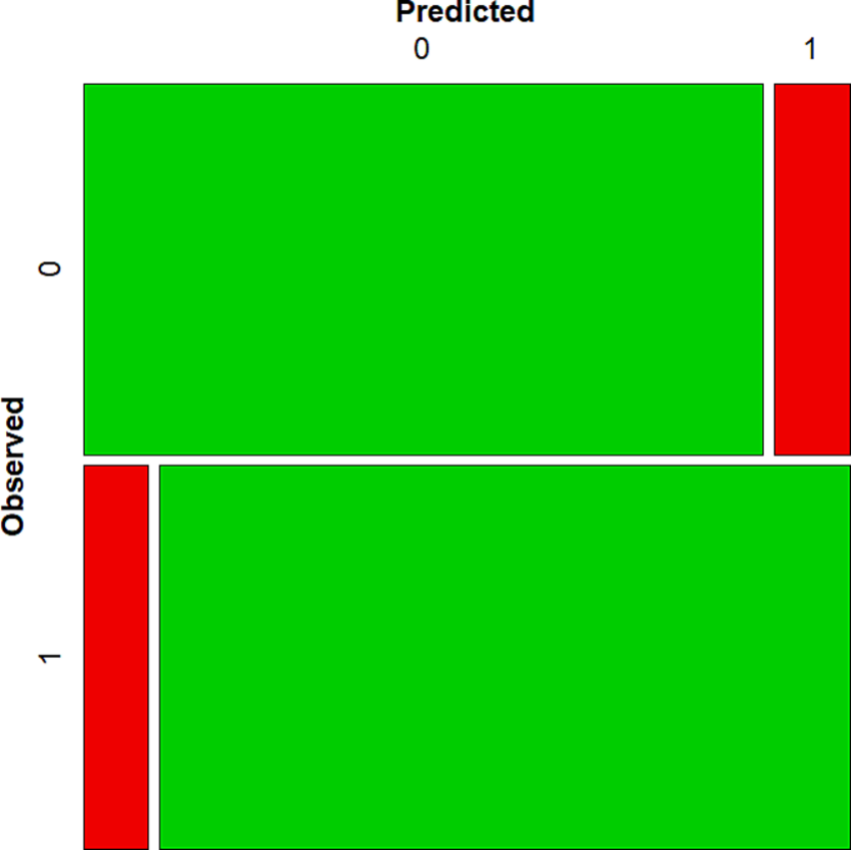
Mosaic plot of observed versus predicted values.

Regarding the model’s predictive ability, the classification matrix values showed a sensitivity index of 91.6% and a specificity index of 90%. And, overall, a correct classification rate of 90.8% (see Table 6; Figure 2). A model with good predictive ability should have high values for both sensitivity and specificity, therefore, the values showed that it is a model with predictive ability. The model’s sensitivity refers to the model’s ability to detect as high flow the cases that actually obtain high flow scores, while the model’s specificity refers to its ability to correctly discriminate cases that do not obtain high flow scores.

**Table 6:** Classification table.

Cat_flow				
		Low	High	Correct percentage
Cat_flow	Low	72	8	90.0
	High	7	76	91.6
Overall percentage				90.8

Sensitivity and specificity indices.

## 4 Discussion and conclusions

In the field of music, an increasing number of studies can be found that deal with musicians’ flow experience (Custodero, 2002, 2005; Fritz and Avsec, 2007; Sinnamon et al., 2012; Fullagar et al., 2013; Marin and Bhattacharya, 2013; Wrigley and Emmerson, 2013; Iusca, 2015; Cohen and Bodner, 2019a,b, 2021; Moral-Bofill et al., 2020b, 2022a,b; Moral-Bofill, 2021, 2022) (see also Chirico et al., 2015; Tan and Sin, 2021). The need for musicians to develop psychological self-regulation skills has been considered, and for training to not only focus exclusively on technical-performance skills (Brodsky, 1996; Williamon, 2004; Clark and Williamon, 2011; Wrigley and Emmerson, 2013; Cohen and Bodner, 2019a; Spahn et al., 2021; Moral-Bofill et al., 2022a,b). In fact, it has been suggested that targeted interventions should be based on skills training related to flow factors, as well as other related factors (Swann, 2016; Moral-Bofill et al., 2022b).

Accumulated research on flow shows that the most universal precondition for promoting flow state is the balance between the perceived challenges in a situation and the individual’s skills to engage. However, the relationships between these variables may be moderated by situational and personal factors (*cf.* Barthelmäs and Keller, 2021). For example, in line with some findings (see Engeser and Rheinberg, 2008; Engeser and Baumann, 2016; Barthelmäs and Keller, 2021) it has been suggested that the optimal challenge concept would predict enjoyment in the context of goal-directed leisure activities, such as sports and games, rather than in contexts where performance outcomes could have greater consequences (Abuhamdeh, 2021). In addition, having clear goals and clear feedback would also be necessary conditions for a person to reach flow state (condition-experience model, Nakamura et al., 2019).

In order to try and detect which predictors can be considered the most suitable for identifying performing musicians with high flow levels during musical performance, the logistic regression (LR) method was applied. The three antecedent variables of the condition-experience model were taken into account, as well as a group of situational and sociodemographic variables that, in general, have shown heterogeneous results in terms of their relationship with the flow experience of performing musicians.

The results of introducing nine variables into the logistic regression analysis that could be related to the performing musicians’ flow state (high flow) showed that only four of them, balance, goals, feedback, and situation, were statistically significant predictors of group membership, while neither gender, age, dedication, musical instrument or style of music were statistically significant predictors. The variables that, according to the condition-experience model (Nakamura et al., 2019), are antecedents for the occurrence of flow state, together with the situation variable, explained almost 80% of the variance of cat_flow. In addition, the classification matrix values showed that the model had a good predictive ability with an overall correct classification rate of 90.8% (see Table 6).

Among the antecedent variables or conditions, balance showed the highest Odds Ratio (OR = 4.394), expressing the extent to which the occurrence of being in a flow state (high flow) increases due to this factor. This result is in line with theory, as it has consistently been shown to be an antecedent of flow in experimental, correlational and regression studies in different settings (Barthelmäs and Keller, 2021; Moneta, 2021). Furthermore, there is evidence with performing musicians that both flow state and musical performance anxiety (MPA) respond to changes in situational contingencies caused by the degree of balance between the challenge of the task and the skills needed to meet that challenge (Fullagar et al., 2013). Balance has also been found to be critical as a mediator between MPA and performance success. Musicians who were more likely to experience MPA reported experiencing an imbalance in their ability to challenge themselves in relation to their abilities (Stocking, 2013).

Similarly, the other two variables considered antecedents in the condition-experience model, goals and feedback, showed a positive association with cat_flow. That is, the clearer the goals and feedback, the higher the odds of obtaining a high flow score. The OR values for both variables were similar (OR = 1.723 for goals and OR = 1.648 for feedback), and express, respectively, to what extent the occurrence of being in flow state (high flow) increases due to each of the two factors. This result is interesting because, although both factors are considered in theory as antecedents of flow, the clear goals factor is understood as the clear understanding of the task structure (or clear task instructions), which will make it possible for the individual to assess their skill-challenge balance level for a specific task, and clear feedback, such as immediate and unambiguous feedback on progress or success in carrying out the activity; and also associated with the task structure being clear. Therefore, they have been considered as intrinsic antecedents to a central antecedent such as the balance between ability and challenge; and this consideration could make the theory more parsimonious by grouping the three antecedents into one (Barthelmäs and Keller, 2021). It is necessary to emphasise that, from this perspective, the goal concept takes on a different meaning from that of goal setting (e.g., Locke and Latham, 2002). However, studies in the field of music have found goal setting to be important in promoting the flow experience, helping to reduce stress and anxiety and improving performance (Fullagar et al., 2013). The LR results in this study showed that both goals and feedback are statistically significant predictors of flow state when controlling the influence of balance. Therefore, they may be important variables for the occurrence of flow state from a goal-setting perspective. The statement My objectives were clearly defined is worded along these lines and suggests more than a clear understanding of a task’s structure or instructions. Furthermore, the condition-experience model (Nakamura et al., 2019) shows that it would be difficult to flow if any of the three conditions were absent (Nakamura et al., 2019). Still, only balance has strong empirical support (Peifer and Engeser, 2021). Therefore, the present results provide empirical evidence about the contribution of goals and feedback in the occurrence of flow state.

On the other hand, of all the situational and sociodemographic variables entered in the regression analysis, only the situation variable was a significant predictor of group membership. That is, the performance situation showed a positive association with cat_flow, specifically, a direct relationship between the free situation and high flow. That is, whether musicians perform in individual or informal situations influences higher odds of being in a flow state (obtaining high flow scores) than if musicians perform in a concert situation (OR = 10.806). This result is also in line with the findings of other studies and with some theoretical considerations (Engeser and Rheinberg, 2008; Engeser and Baumann, 2016; Abuhamdeh, 2021; Barthelmäs and Keller, 2021). Although the most universal precondition for triggering the flow response is considered to be the balance between skills and challenge, there are situational (and personal) factors that would moderate the relationship between skills and challenge (*cf.* Barthelmäs and Keller, 2021). In those performance situations where performance outcomes do not lead to (or are not interpreted with) possible negative consequences, it would be a factor that would increase the odds of high flow occurring (controlling the influence of the rest of the variables introduced in the model, that is, the antecedent variables). This finding is very interesting in order to continue finding strategies to help musicians enjoy concerts. The point is that, although all three antecedents have been optimised, the perception of the situation they have to face may or may not promote greater enjoyment and possibly less MPA. However, the confidence interval of the OR is quite wide (between 2.120 and 55.097), this variability means that its influence can vary greatly depending on other factors.

From our point of view, the implications of these results for the training of performing musicians are particularly relevant and, if taken into account, could contribute to improving the expressive capacity and performing experience of musicians. Firstly, regardless of variables such as gender, age, type of musical instrument played, musical style performed, or relationship with the musical activity, the more the performing musicians appreciate that there is a better balance between their skills and the challenge they face, the greater their opportunity to experience flow state (high flow). Therefore, during performing musicians’ preparation, it would be key to bear in mind the importance of this variable, in music schools of any level, it can be neglected when issues unrelated to the musicians’ training needs are prioritised. This can happen, for example, when musicians are forced to prepare a repertoire without sufficient time; when certain instrumental musical skills are (wrongly) considered sufficient in contexts that require the acquisition of other skills and, therefore, extra preparation; when a musician is asked to perform a piece or repertoire in which they feel uncomfortable because they sense that their skills are still inadequate; when no attention is paid to a proper sequencing of the skills to be developed; or when not enough time is given to establish the skills.

Secondly, goals and feedback are also variables that contribute to increasing the opportunity to experience flow state. For example, setting clear goals during the study, practise or performance, what you want to achieve in terms of specific actions, including actions aimed at coping with the performance. It is important to define realistic goals or sub-goals, clear objectives for performance and professional development. As Csikszentmihalyi (1990) comments, in music, as in other creative activities, goals are not always clearly defined beforehand. However, it is necessary to have internalised the criteria in order to know what is right and what is not. Hence the importance of clear feedback. In order to help focus attention on the goals, clear feedback is necessary to help know whether one is on track to achieve those goals. Therefore, what is communicated to the student or performer during their learning, lessons, rehearsals, tests or public performances will be key. The type of feedback that will provide valuable information will be that which helps the musician become more competent in specific tasks; to focus on and work on them; to activate curiosity and interest in moving toward clear goals and to reinforce confidence. Furthermore, this will also contribute to creating a balanced view of oneself (Jackson and Csikszentmihalyi, 1999).

Finally, when musicians perform in situations they consider informal or without an audience, the odds of experiencing flow state increase, so when a performance situation is perceived by musicians as less compromising (less threatening), or without possible negative consequences. Given this outcome, it may be key to keep in mind the intrinsic reasons why performers dedicate themselves to music. Music is considered an autotelic activity. Namely, it has the potential to maximise immediate intrinsic rewards to the musician, and therefore promote flow state. According to Sinnamon (2020), optimal experience and peak performance usually open happen when intrinsic motivation is present, when the internal contentment accompanying the action is present, and the performer is fully involved in the music. Furthermore, when musicians are faced with an optimal challenge, it will be essential that they feel totally confident in their ability to play and to carry out the challenge successfully. Therefore, preparation will be essential. Careful and extensive preparation (musically and mentally) is what encourages musicians facing a sense of optimal challenge (or risk), to experience confidence, to be able to let go and free themselves from fear of failure, worries about making mistakes, memory failure, that their performance will not be liked by the audience or that they will not be called back to play. The result of taking the risk and letting it go can be the optimal experience, flow state (Sinnamon, 2020). Similarly, another strategy that musicians (and teachers) might consider for concert or assessment situations is to reflect on whether they can select repertoire in which they perceive their abilities exceed the demands.

On the other hand, while careful preparation would be essential, perfectionism could be counterproductive. Promoting balanced and adaptive thinking about their own activity would help to prevent its negative consequences. Musicians work hard to express a “perfect” sound or musical idea in real life, although this is a motivating purpose, it is important that they can assess the extent to which their expectations are reasonable (e.g., “I cannot play if I do not get this passage with perfect dynamics”). Furthermore, what often underlies this need for perfection is the belief that musicians must appear perfect all the time. They must show that they are able to play easily, as if they cannot have a hard or difficult time, or show that they are human, and can make mistakes. In general, perfectionist cognitions include setting unrealistically high performance standards or the constant need to be perceived as flawless (Frost et al., 1990; Flett et al., 2002). Therefore, behind the constant effort to make the music perfect, there is often a major concern about mistakes and the need to avoid a subjective experience of failure. In fact, perfectionism has been related to MPA (see Dobos et al., 2019) and, specifically, maladaptive perfectionism has been related to psychological rigidity in music students, which in turn was related to higher levels of MPA and showed an indirect effect on students’ flow experiences when performing (Osborne et al., 2021). In short, freeing oneself from the fear of failure, from the fear of failing, would be one of the strategies that could contribute to reaching flow state because it would transform any situation, including formal or evaluative public exposure, into a truly autotelic activity.

Regarding the rest of the variables that have not shown predictive ability to identify musicians who experience high flow, some considerations need to be taken into account. The logic behind LR is to preserve the independent variables that contain relevant information and, at the same time, to get rid of those that are redundant with respect to those that remain in the model. This procedure is exclusively statistical in nature; it runs according to programmable algorithms in which, once the initial set of variables has been chosen, the researchers’ theoretical judgements do not intervene (Silva and Barroso, 2004). It is therefore important to introduce the variables that theory identifies as important into the logistic regression analysis. It should be noted that in this study the initial choice was guided by the findings of previous studies and by theory. In short, given the three relevant variables of the condition-experience model, together with the situation variable, the rest of the variables that were introduced did not show predictive ability; statistically speaking, their information led to the variables that did show this ability. But that does not mean that the differences in flow found by some studies with these variables (gender, age, type of musical instrument, musical style, or relationship with musical activity) do not exist. These differences may exist, but there may be confounding variables. For example, perhaps the differences that have been found based on gender actually relate to differences between men and women regarding their ability to optimally adjust challenges and skills, or their ability to set clear goals. Or perhaps the differences that have been found between musicians of different styles actually have to do with the type of situation in which they usually play. In any case, a musician will increase their chances of experiencing flow state if they perceive a balance between the skills they have and the challenge they are facing, if they have clearly defined goals and clear feedback, and perceive the musical activity as a genuine autotelic activity, whereas it will have little to do with whether they are male or female, what musical instrument they play, the style of music, whether they are a student, amateur or professional, or their age.

Before concluding, it is necessary to comment on some limitations. One limitation of this study is related to the data collection process, specifically, not having extracted a simple random sample in order to carry out the logistic regression analysis that guarantees the assumption of independence between observations. Another limitation concerns the predictive model found. Although relevant variables were introduced according to the theory, there are other factors (especially personality characteristics) that could also have been relevant in the model, therefore, the repertoire of variables that have remained in the model could have changed. A future line of research could be to collect data on these variables in order to introduce them into the regression model and see which ones show predictive ability and which do not. Another line of research would be to clarify whether there are confounding variables that prevent us from focusing on the relevant issues to improve the musicians’ performance experience. Furthermore, the results of this study also raise interesting questions for music schools. For example, questions could be asked about whether assessment situations contribute to developing the performing musician’s performance skills: is it educational for musicians to experience assessment situations as threatening? Could other efficient assessment strategies be proposed to enhance musicians’ abilities (instead of weakening them) and their performance skills?.

## Data availability statement

The original contributions presented in the study are included in the article/Supplementary material, further inquiries can be directed to the corresponding author.

## Ethics statement

The studies involving humans were approved by the Department of Behavioural Sciences Methodology of the Faculty of Psychology at the National University of Distance Education (UNED, in its Spanish acronym). Furthermore, the study was conducted in accordance with the latest Helsinki Declaration [World Medical Association (WMA), 2022]. The studies were conducted in accordance with the local legislation and institutional requirements. Written informed consent for participation was not required from the participants or the participants’ legal guardians/next of kin in accordance with the local legislation and institutional requirement. Moreover, participation consisted of completing the form that was programmed using the Google Forms tool, and recipients were informed that participation was anonymous and voluntary. Written informed consent was obtained from the individual(s) for the publication of any potentially identifiable images or data included in this article.

## Author contributions

LM-B: Conceptualization, Data curation, Formal analysis, Investigation, Methodology, Writing – original draft, Writing – review & editing. AL: Supervision, Writing – review & editing. MP-L: Supervision, Writing – review & editing.

## References

[ref1] Abuhamdeh S. (2020). Investigating the “flow” experience: key conceptual and operational issues. Front. Psychol. 11:158. doi: 10.3389/fpsyg.2020.00158, PMID: 32116954 PMC7033418

[ref9001] Abuhamdeh S. (2021). “Flow theory and cognitive evaluation theory: Two sides of the same coin?.,” in Advances in Flow Research. eds. Peifer C. Engeser S. (Springer, Cham).

[ref2] Abuhamdeh S. Csikszentmihalyi M. (2012). The importance of challenge for the enjoyment of intrinsically motivated, goal-directed activities. Personal. Soc. Psychol. Bull. 38, 317–330. doi: 10.1177/0146167211427147, PMID: 22067510

[ref5] Bakker A. B. (2005). Flow among music teachers and their students: the crossover of peak experiences. J. Vocat. Behav. 66, 26–44. doi: 10.1016/j.jvb.2003.11.001

[ref6] Barthelmäs M. Keller J. (2021). “Antecedents, boundary conditions and consequences of flow” in Advances in flow research. eds. Peifer C. Engeser S. (Cham: Springer), 71–107.

[ref7] Baumann N. Lürig C. Engeser S. (2016). Flow and enjoyment beyond skill-demand balance: the role of game pacing curves and personality. Motiv. Emot. 40, 507–519. doi: 10.1007/s11031-016-9549-7

[ref8] Bricteux C. Navarro J. Ceja L. Fuerst G. (2017). Interest as a moderator in the relationship between challenge/skills balance and flow at work: an analysis at within-individual level. J. Happiness Stud. 18, 861–880. doi: 10.1007/s10902-016-9755-8

[ref9] Brodsky W. (1996). Music performance anxiety reconceptualized: a critique of current research practices and findings. Med. Probl. Perform. Art. 11, 88–98.

[ref10] Bryce J. Haworth J. (2002). Wellbeing and flow in sample of male and female office workers. Leis. Stud. 21, 249–263. doi: 10.1080/0261436021000030687

[ref11] Bullerjahn C. Dziewas J. Hilsdorf M. Kassl C. Menze J. Gembris H. (2020). Why adolescents participate in a music contest and why they practice – the influence of incentives, flow, and volition on practice time. Front. Psychol. 11:561814. doi: 10.3389/fpsyg.2020.561814, PMID: 33192831 PMC7652895

[ref13] Byrne C. MacDonald R. Carlton L. (2003). Assessing creativity in musical compositions: flow as an assessment tool. Br. J. Music Educ. 20, 277–290. doi: 10.1017/S0265051703005448

[ref14] Carli M. Fave A. D. Massimini F. (1988). “The quality of experience in the flow channels: comparison of Italian and US students” in Optimal experience: Psychological studies of flow in consciousness. eds. Csikszentmihalyi M. Csikszentmihalyi I. S. (Cambridge: Cambridge University Press), 288–306.

[ref16] Ceja L. Navarro J. (2012). ‘Suddenly I get into the zone’: examining discontinuities and nonlinear changes in flow experiences at work. Hum. Relat. 65, 1101–1127. doi: 10.1177/0018726712447116

[ref17] Chirico A. Serino S. Cipresso P. Gaggioli A. Riva G. (2015). When music “flows”. State and trait in musical performance, composition and listening: a systematic review. Front. Psychol. 6:906. doi: 10.3389/fpsyg.2015.00906, PMID: 26175709 PMC4485232

[ref18] Clark T. Lisboa T. Williamon A. (2014). An investigation into musicians’ thoughts and perceptions during performance. Res. Stud. Music Educ. 36, 19–37. doi: 10.1177/1321103X14523531

[ref19] Clark T. Williamon A. (2011). Evaluation of a mental skills training program for musicians. J. Appl. Sport Psychol. 23, 342–359. doi: 10.1080/10413200.2011.574676

[ref20] Cohen S. Bodner E. (2019a). The relationship between flow and music performance anxiety amongst professional classical orchestral musicians. Psychol. Music 47, 420–435. doi: 10.1177/0305735618754689

[ref21] Cohen S. Bodner E. (2019b). Music performance skills: a two-pronged approach–facilitating optimal music performance and reducing music performance anxiety. Psychol. Music 47, 521–538. doi: 10.1177/0305735618765349

[ref22] Cohen S. Bodner E. (2021). Flow and music performance anxiety: the influence of contextual and background variables. Music. Sci. 25, 25–44. doi: 10.1177/1029864919838600

[ref23] Crust L. Swann C. (2013). The relationship between mental toughness and dispositional flow. Eur. J. Sport Sci. 13, 215–220. doi: 10.1080/17461391.2011.635698

[ref24] Csikszentmihalyi M. (1975). Beyond boredom and anxiety. San Francisco: Jossey-Bass.

[ref25] Csikszentmihalyi M. (1988). “The future of flow” in Optimal experience: Psychological studies of flow in consciousness. eds. Csikszentmihalyi M. Csikszentmihalyi I. S. (New York: Cambridge University Press), 364–383.

[ref26] Csikszentmihalyi M. (1990). Flow: The psychology of optimal experience. New York: Harper & Row.

[ref27] Csikszentmihalyi M. (1996). Creativity: The work and lives of 91 eminent people. New York: Harper Collins.

[ref28] Csikszentmihalyi M. LeFevre J. (1989). Optimal experience in work and leisure. J. Pers. Soc. Psychol. 56, 815–822. doi: 10.1037/0022-3514.56.5.815 2724069

[ref29] Csikszentmihalyi M. Rich G. (1998). “Musical improvisation: a systems approach” in Creativity in performance. ed. Sawyer K. (Greenwich, CT: Ablex), 43–66.

[ref30] Custodero L. A. (2002). Seeking challenge, finding skill: flow experience and music education. Arts Educ. Policy Rev. 103, 3–9. doi: 10.1080/10632910209600288

[ref31] Custodero L. A. (2005). Observable indicators of flow experience: a developmental perspective on musical engagement in young children from infancy to school age. Music. Educ. Res. 7, 185–209. doi: 10.1080/14613800500169431

[ref32] Delle Fave A. Massimini F. Bassi M. (2011). Psychological selection and optimal experience across cultures: Social empowerment through personal growth (Vol. 2). Springer Science & Business Media, New York.

[ref33] Dobos B. Piko B. F. Kenny D. T. (2019). Music performance anxiety and its relationship with social phobia and dimensions of perfectionism. Res. Stud. Music Educ. 41, 310–326. doi: 10.1177/1321103X18804295

[ref34] Eisenberger R. Jones J. R. Stinglhamber F. Shanock L. Randall A. T. (2005). Flow experiences at work: for high need achievers alone? J. Organ. Behav. 26, 755–775. doi: 10.1002/job.337

[ref35] Engeser S. Baumann N. (2016). Fluctuation of flow and affect in everyday life: a second look at the paradox of work. J. Happiness Stud. 17, 105–124. doi: 10.1007/s10902-014-9586-4

[ref36] Engeser S. Rheinberg F. (2008). Flow, performance, and moderators of challenge-skill balance. Motiv. Emot. 32, 158–172. doi: 10.1007/s11031-008-9102-4

[ref37] Flett G. L. Hewitt P. L. Oliver J. M. Macdonald S. (2002). “Perfectionism in children and their parents: a developmental analysis” in Perfectionism: Theory, research, and treatment. eds. Flett G. L. Hewitt P. L. (American Psychological Association, Washington, DC), 89–132.

[ref38] Fritz B. S. Avsec A. (2007). The experience of flow and subjective well-being of music students. Horizons psychol. 16, 5–17.

[ref39] Frost R. O. Marten P. Lahart C. Rosenblate R. (1990). The dimensions of perfectionism. Cogn. Ther. Res. 14, 449–468. doi: 10.1007/BF01172967

[ref40] Fullagar C. J. Knight P. A. Sovern H. S. (2013). Challenge/skill balance, flow, and performance anxiety. Appl. Psychol. 62, 236–259. doi: 10.1111/j.1464-0597.2012.00494.x

[ref41] Habe K. Biasutti M. Kajtna T. (2019). Flow and satisfaction with life in elite musicians and top athletes. Front. Psychol. 10:698. doi: 10.3389/fpsyg.2019.00698, PMID: 30984086 PMC6450199

[ref42] Habe K. Biasutti M. Kajtna T. (2021). Wellbeing and flow in sports and music students during the COVID-19 pandemic. Think. Skills Creat. 39:100798. doi: 10.1016/j.tsc.2021.100798, PMID: 33589864 PMC7874927

[ref43] Harmat L. de Manzano Ö. Ullén F. (2021). “Flow in music and arts” in Advances in flow research. eds. Peifer C. Engeser S. (Cham: Springer), 377–391.

[ref44] Heller K. Bullerjahn C. von Georgi R. (2015). The relationship between personality traits, flow-experience, and different aspects of practice behavior of amateur vocal students. Front. Psychol. 6:1901. doi: 10.3389/fpsyg.2015.01901, PMID: 26733904 PMC4685080

[ref45] Iusca D. (2015). The relationship between flow and music performance level of undergraduates in exam situations: the effect of musical instrument. Procedia Soc. Behav. Sci. 177, 396–400. doi: 10.1016/j.sbspro.2015.02.376

[ref46] Jackman P. C. Hawkins R. M. Crust L. Swann C. (2019). Flow states in exercise: a systematic review. Psychol. Sport Exerc. 45:101546. doi: 10.1016/j.psychsport.2019.101546

[ref47] Jackson S. Csikszentmihalyi M. (1999). Flow in sport: The keys to optimal experiences and performances. Champaign, IL: Human Kinetics.

[ref48] Jackson S. A. Eklund R. C. (2002). Assessing flow in physical activity: the flow state scale-2 and dispositional flow scale-2. J. Sport Exerc. Psychol. 24, 133–150. doi: 10.1123/jsep.24.2.133

[ref49] Jackson S. A. Eklund R. C. (2004). The flow scale manual. Morgantown, WV: Fitness Information Technology.

[ref50] Keller J. Bless H. (2008). Flow and regulatory compatibility: an experimental approach to the flow model of intrinsic motivation. Personal. Soc. Psychol. Bull. 34, 196–209. doi: 10.1177/0146167207310026 18212330

[ref51] Keller J. Blomann F. (2008). Locus of control and the flow experience: an experimental analysis. Eur. J. Personal. 22, 589–607. doi: 10.1002/per.692

[ref52] Kenny D. T. Ackermann B. (2016). “Optimizing physical and psychological health in performing musicians” in The Oxford handbook of music psychology. eds. Hallam S. Cross I. Thaut M.. 2nd ed (Oxford: Oxford University Press), 390–400.

[ref53] Kirchner J. M. Bloom A. J. Skutnick-Henley P. (2008). The relationship between performance anxiety and flow. Med. Probl. Perform. Art. 23, 59–65. doi: 10.21091/mppa.2008.2012

[ref54] Kuhnle C. Hofer M. Kilian B. (2012). Self-control as predictor of school grades, life balance, and flow in adolescents. Br. J. Educ. Psychol. 82, 533–548. doi: 10.1111/j.2044-8279.2011.02042.x, PMID: 23025391

[ref55] Kutepova-Bredun V. (2018). The properties of the flow state and the personal features of the professional and amateur musicians. J. Psychol. Res. 24, 88–95.

[ref56] Lefevre J. (1988). “Flow and the quality of experience during work and leisure” in Optimal experience: Psychological studies of flow in consciousness. eds. Csikszentmihalyi M. Csikszentmihalyi I. S. (Cambridge: Cambridge University Press), 307–318.

[ref57] Locke E. A. Latham G. P. (2002). Building a practically useful theory of goal setting and task motivation: a 35-year odyssey. Am. Psychol. 57, 705–717. doi: 10.1037/0003-066X.57.9.705, PMID: 12237980

[ref58] MacDonald R. Byrne C. Carlton L. (2006). Creativity and flow in musical composition: an empirical investigation. Psychol. Music 34, 292–306. doi: 10.1177/0305735606064838

[ref59] Marin M. M. Bhattacharya J. (2013). Getting into the musical zone: trait emotional intelligence and amount of practice predict flow in pianists. Front. Psychol. 4:853. doi: 10.3389/fpsyg.2013.00853, PMID: 24319434 PMC3837225

[ref60] Moneta G. B. (2021). “On the conceptualization and measurement of flow” in Advances in flow research. eds. Peifer C. Engeser S. (Cham: Springer), 31–69.

[ref61] Moral-Bofill L. (2021). “Desarrollo de la respuesta de Fluidez (Flow). En A. LópezdelaLlave y M. C. Pérez-Llantada” in Psicología y artes escénicas (Madrid: Dykinson), 205–228.

[ref62] Moral-Bofill L. (2022). El estado de fluidez en intérpretes de música: evaluación y condiciones para su desarrollo. Doctoral dissertation. UNED. Universidad Nacional de Educación a Distancia. Available at: http://e-spacio.uned.es/fez/view/tesisuned:ED-Pg-PsiSal-Lmoral

[ref63] Moral-Bofill L. López de la Llave A. Pérez-Llantada M. C. (2019). Estudio del estado de Flow en intérpretes de música. En las actas del VI Congreso de Conservatorios Superiores de Música (CONSMU VI). CSM de Canarias, sede de Tenerife. doi: 10.13140/RG.2.2.33574.88648,

[ref64] Moral-Bofill L. López de la Llave A. Pérez-Llantada M. C. (2020a). Relationships between high ability (gifted) and flow in music performers: pilot study results. Sustainability 12:4289. doi: 10.3390/su12104289

[ref65] Moral-Bofill L. López de la Llave A. Pérez-Llantada M. C.. (2022a). Influencia de las intervenciones psicológicas y/o corporales en la Fluidez y la Ansiedad Escénica Musical de los intérpretes de música. Blanco-Piñeiro M Ya Zubeldia Echeberria Llave Rodríguez López de la (Eds.) Investigaciones y experiencias profesionales en Psicología de las Artes Escénicas. Editorial UNED

[ref66] Moral-Bofill L. López de la Llave A. Pérez-Llantada M. C. Holgado-Tello F. P. (2020b). Adaptation to Spanish and psychometric study of the flow state Scale-2 in the field of musical performers. PLoS One 15:e0231054. doi: 10.1371/journal.pone.0231054, PMID: 32240253 PMC7117763

[ref67] Moral-Bofill L. López de la Llave A. Pérez-Llantada M. Holgado-Tello F. P. (2022b). Development of flow state self-regulation skills and coping with musical performance anxiety: design and evaluation of an electronically implemented psychological program. Madrid. Front. Psychol. 13:899621. doi: 10.3389/fpsyg.2022.899621, PMID: 35783805 PMC9248863

[ref68] Mosing M. A. Pedersen N. L. Cesarini D. Johannesson M. Magnusson P. K. Nakamura J.. (2012). Genetic and environmental influences on the relationship between flow proneness, locus of control and behavioral inhibition. PLoS One 7:e47958. doi: 10.1371/journal.pone.0047958, PMID: 23133606 PMC3487896

[ref69] Nakamura J. Tse D. C. K. Shankland S. (2019). “Flow: the experience of intrinsic motivation” in The Oxford handbook of human motivation. ed. Ryan R. M. (New York, Oxford University Press), 169–185.

[ref70] Norsworthy C. Gorczynski P. Jackson S. A. (2017). A systematic review of flow training on flow states and performance in elite athletes. Grad. J. Sport, Exercise Physical Educ. Res. 6, 16–28.

[ref72] Osborne M. Roman J. Juncos D. Zenobi D. (2021). Psychological inflexibility and its relation to performance anxiety, flow, and perfectionism in university musicians. Conference: Association for Contextual Behavioral Science [internet]. Melbourne: University of Melbourne.

[ref73] Ottiger B. Van Wegen E. Keller K. Nef T. Nyffeler T. Kwakkel G.. (2021). Getting into a “flow” state: a systematic review of flow experience in neurological diseases. J. Neuroeng. Rehabil. 18, 65–21. doi: 10.1186/s12984-021-00864-w, PMID: 33879182 PMC8059246

[ref74] Peifer C. Engeser S. (2021). “Theoretical integration and future lines of flow research” in Advances in flow research (Cham: Springer), 417–439.

[ref75] Peifer C. Wolters G. (2021). “Flow in the context of work” in Advances in flow research. eds. Peifer C. Engeser S. (Cham: Springer), 287–321.

[ref77] Rathunde K. Csikszentmihalyi M. (2005). Middle school Students' motivation and quality of experience: a comparison of Montessori and traditional school environments. Am. J. Educ. 111, 341–371. doi: 10.1086/428885

[ref78] Schallberger U. Pfister R. (2001). Flow-Erleben in Arbeit und Freizeit. Eine Untersuchung zum “paradox der Arbeit” mit der experience sampling method (ESM) [flow experiences in work and leisure. An experience sampling study about the paradox of work]. Zeitschrift für Arbeitsund Organisationspsychologie 45, 176–187. doi: 10.1026//0932-4089.45.4.176

[ref80] Silva L. C. Barroso I. M. (2004). Regresión logística. La Muralla. Madrid, Spain

[ref81] Sinnamon S. (2020). Achieving peak performance in music: psychological strategies for optimal flow. Routledge. London

[ref82] Sinnamon S. Moran A. O’Connell M. (2012). Flow among musicians: measuring peak experiences of student performers. J. Res. Music. Educ. 60, 6–25. doi: 10.1177/0022429411434931

[ref83] Spahn C. Krampe F. Nusseck M. (2021). Live music performance: the relationship between flow and music performance anxiety. Front. Psychol. 12:725569. doi: 10.3389/fpsyg.2021.725569, PMID: 34899468 PMC8655696

[ref84] Stocking B. H. (2013). Music performance anxiety and dispositional flow in predicting audition success in amateur percussionists. (Master's Thesis). Knoxville: University of Tennessee.

[ref85] Swann C. (2016). “Flow in sport” in The flow experience: Empirical research and applications. eds. Harmat L. Ørsted Andersen F. Ullén F. Wright J. Sadlo G. (Cham: Springer), 51–64.

[ref86] Tan L. Sin H. X. (2021). Flow research in music contexts: a systematic literature review. Music. Sci. 25, 399–428. doi: 10.1177/1029864919877564

[ref87] Teng C. I. (2011). Who are likely to experience flow? Impact of temperament and character on flow. Personal. Individ. Differ. 50, 863–868. doi: 10.1016/j.paid.2011.01.012

[ref88] Triberti S. Di Natale A. F. Gaggioli A. (2021). “Flowing technologies: the role of flow and related constructs in human-computer interaction,” in Advances in Flow Research. eds. Peifer C. Engeser S. (Springer, Cham).

[ref89] Ullén F. de Manzano Ö. Almeida R. Magnusson P. K. Pedersen N. L. Nakamura J.. (2012). Proneness for psychological flow in everyday life: associations with personality and intelligence. Personal. Individ. Differ. 52, 167–172. doi: 10.1016/j.paid.2011.10.003

[ref92] Williamon A. (Ed.). (2004). Musical excellence: Strategies and techniques to enhance performance. Oxford University Press, Madison.

[ref93] Woody R. H. McPherson G. E. (2010). “Emotion and motivation in the lives of performers” in Handbook of music and emotion: theory, research, applications. eds. Juslin P. N. Sloboda J. A. (Oxford: Oxford University Press), 401–424.

[ref94] World Medical Association (WMA) (2022). Declaración de Helsinki para la investigación con seres humanos. Available at: https://www.wma.net/es/policies-post/declaracion-de-helsinki-de-la-amm-principios-eticos-para-las-investigaciones-medicas-en-seres-humanos/ (Accessed August 27, 2020).

[ref95] Wrigley W. J. Emmerson S. B. (2013). The experience of the flow state in live music performance. Psychol. Music 41, 292–305. doi: 10.1177/0305735611425903

